# GRANDPARENTS’ PERSPECTIVES ON A MULTIGENERATIONAL DIGITAL HEALTH PHYSICAL ACTIVITY INTERVENTION

**DOI:** 10.1093/geroni/igz038.2487

**Published:** 2019-11-08

**Authors:** Marissa Kobayashi, Rafael O Leite, Isabella Lugo, Rachel Wetstone, Dario Vanegas, Sara M St George, Sara J Czaja

**Affiliations:** 1 University of Miami, Miami, Florida, United States; 2 University of Miami Miller School of Medicine, Miami, Florida, United States; 3 Weill Cornell Medicine, New York, New York, United States

## Abstract

Studies examining associations among grandparents’ involvement and grandchildren’s lifestyle behaviors have been largely mixed or negative highlighting the need for interventions that help grandparents promote grandchildren’s healthy behaviors. The current study explored older adults’ interest in participating in a digital intervention with their grandchildren. As part of the intervention, grandparents and grandchildren would engage in weekly walks and use a mobile application to track their steps, photos and conversations. Twelve grandparents (63±6.5yrs; 75% female; 50% Hispanic) participated in qualitative interviews. Researchers asked open-ended questions to assess grandparents’ relationships with their grandchildren, motivation to participate, and feedback on prototypes. A 10-item systems usability questionnaire was also administered. Three researchers independently analyzed interview transcripts using a rapid assessment approach and reached consensus on key themes. Grandparents described having positive relationships with their grandchildren and used texts to schedule time with them; family dynamics (conflicts, divorce) influenced the amount of time they spent together. Grandparents’ motivation for participating in the intervention included the opportunity to enhance their relationship with their grandchild and improve their own health. Grandparents noted weekly walks would feasibly occur on the weekends given their grandchildren’s competing activities. They were receptive to proposed weekly session topics (e.g., sports/hobbies, ancestry, humor) and suggested other topics to discuss during weekly walks, such as faith/religion, morality, safety, and nutrition. They strongly agreed or agreed that they would use the prototypes frequently and found them easy to use (83% and 92%, respectively). Results from this study will inform the next iteration of intervention prototypes.

